# Meeting report: a close look at oral biofilms and microbiomes

**DOI:** 10.1038/s41368-018-0030-1

**Published:** 2018-08-15

**Authors:** Xin Xu, Feng Chen, Zhengwei Huang, Lvyan Ma, Li Chen, Yaping Pan, Jian Xu, Syngcuk Kim, Denis Kinane, Hyun Koo, Xuedong Zhou

**Affiliations:** 1 State Key Laboratory of Oral Diseases & National Clinical Research Center for Oral Diseases & Department of Cariology and Endodontics, West China Hospital of Stomatology, Sichuan University, Chengdu, China; 2 Central Laboratory, Peking University School and Hospital of Stomatology, Beijing, China; 3 Department of Endodontics, Ninth People’s Hospital, Shanghai Jiao Tong University School of Medicine, Shanghai, China; 4 State Key Laboratory of Microbial Resources Institute of Microbiology, Chinese Academy of Sciences, Beijing, China; 5 Key Laboratory of Medical Molecular Virology, Ministry of Education and Health, Fudan University, Shanghai, China; 6 Department of Periodontics, School of Stomatology, Chinese Medical University, Shenyang, China; 7 Qingdao Institute of Bioenergy and Bioprocess Technology, Chinese Academy of Sciences, Qingdao, China; 8 Department of Endodontics, School of Dental Medicine, University of Pennsylvania, Philadelphia, PA USA; 9 Department of Periodontics, School of Dental Medicine, University of Pennsylvania, Philadelphia, PA USA; 10 Divisions of Pediatric Dentistry and Community Oral Health, Department of Orthodontics, School of Dental Medicine, University of Pennsylvania, Philadelphia, PA USA

## Abstract

The “Biofilms, Microbiomes and Oral Diseases: Challenges and Future Perspectives” symposium jointly organized by Penn Dental Medicine and West China School of Stomatology was held on 30 September 2017 at Penn Wharton China Center (PWCC) in Beijing, China. The topics included the pathogenicity of oral biofilms, novel strategies for the control of biofilm-related diseases, oral microbiome and single-cell approaches, and the link between oral diseases and overall health. Researchers from a number of disciplines, representing institutions from China and Penn Dental Medicine, gathered to discuss advances in our understanding of biofilms, as well as future directions for the control of biofilm-related oral and systemic diseases.

Biofilms are generally defined as structured communities of microorganisms that are bound to each other and to surfaces. These cells are often embedded within a matrix of extracellular polymeric substances as well.^1–3^ Biofilm formation is an essential virulence trait associated with many human infections.^4,5^ In the mouth, polymicrobial biofilms formed on tooth and mucosal surfaces, implants, or dental materials can cause a wide range of oral diseases and complications.^6–9^ Advances in DNA- and RNA-sequencing reveal the complexity of the biofilm microbiota at different oral sites.^10–12^ Powered by the recent advancements in high-throughput metagenomics and other -omics techniques, approximately 700 species of microbes have been identified in the oral cavity.^13–15^ Parallel to this, the importance of the matrix for the spatial organization of microbial communities and their interactions, and creation of localized microenvironments has been increasingly recognized.^1,2^ The interactions between the local microbiota and the host factors ultimately mediate the transition from health to disease.^6,9,14^ Changes in host diet such as excessive carbohydrate consumption promote accumulation of a matrix-rich environment and acid-producing organisms that trigger ecological changes towards cariogenic microbiota, leading to the onset of dental caries.^16–19^ Keystone pathogens residing in the subgingival environment can modulate the host immune response and polymicrobial balance, resulting in a dysbiotic community that causes periodontitis.^20^ Furthermore, components of the microbiome in the various oral biofilms have also been associated with systemic diseases, including diabetes,^21^ respiratory,^22–24^ cardiovascular,^25^ and inflammatory bowel diseases,^26,27^ among others. Advancements through multidisciplinary and collaborative efforts may be key to further understanding the pathogenic mechanisms, and lead to more effective and precise therapeutic approaches. Given the importance and the global impact of biofilms and microbiomes on oral diseases, we have jointly organized the symposium titled “Biofilms, Microbiomes and Oral Diseases: Challenges and Future Perspectives”.

Penn Dental Medicine is among the oldest university-affiliated dental institutions in the USA, and it is generally recognized as a leader in the education and training of clinicians, researchers, and academicians that continue to advance the field of dental medicine. This year’s symposium marked the second collaborative research symposium of the Penn China Research & Engagement Fund (Penn CREF). Penn Dental Medicine, one of the first recipients of Penn CREF, has aimed to support the development of high-level research symposia on bone, biofilm, and stem cells, as well as the expansion of conferences on the delivery of dental care to China’s vast population in recent years. West China School of Stomatology is widely regarded as the birthplace of China’s modern dentistry and is affiliated with the State Key Laboratory of Oral Diseases (SKLOD), a unique state key laboratory in stomatology supported by the Ministry of Science and Technology of China. We are extremely proud to have had Penn Dental Medicine and SKLOD jointly organize this year’s symposium on oral biofilm on 30 September at Penn Wharton China Center (PWCC) in Beijing, China. This year’s symposium provided a platform for discussion and collaboration between researchers who are at the forefront studying mechanisms of biofilm virulence, polymicrobial interactions and seeking novel therapeutic approaches. In addition, junior faculty, postdoctoral fellows, graduate students, and research investigators gathered for a day of exchange and collaboration with colleagues across disciplines. Lively discussions took place throughout the day, and this meeting report highlights some of the major findings and ideas that emerged during the symposium.

## Microbial biofilms and oral and systemic diseases

### Microbial biofilms and oral diseases

Oral microbial biofilms are comprised of species that span the continuum from commensal to pathogenic. The pathogens are best known for their ability to cause oral infectious diseases, such as dental caries and periodontitis. Dental caries is one of the most common oral infectious diseases initiated by oral biofilm, which is characterized by complex interactions that occur between specific oral microorganisms.^9^ Bacteria in dental plaque communicate with each other through a variety of chemical signaling molecules. One means by which bacterial cells communicate is through quorum sensing (QS).^28–31^ This process involves the production and secretion of a signaling molecule called an autoinducer (AI).^31^ Both gram-negative and gram-positive bacteria can use an autoinducer-2 (AI-2) molecule,^31–33^ which is synthesized by the LuxS enzyme.^29^ LuxS-based signaling has been identified to regulate a broad range of bacterial activities by using the *luxS* mutant strain.^34,35^ Professor Zhengwei Huang’s group from the Ninth People’s Hospital, Shanghai Jiao Tong University School of Medicine provided updates regarding AI-2 regulatory mechanisms. Their group demonstrated that the autoinducer-2-mediated, quorum-sensing system, rather than the activated methyl cycle, was responsible for *luxS* mutant regulation by constructing a quorum-sensing and metabolic independent system.

Development of root caries is similar to coronal caries because the etiology involves predominance by aciduric bacteria. For many years, the *Actinomyces*, *Streptococcus*, *and Lactobacillus* species were considered to be the main agents of root caries.^36,37^ Work by Professor Koo from University of Pennsylvania School of Dental Medicine has shown a strong symbiosis between *Streptococcus mutans* and *Candida albicans*, leading to an increase in biofilm mass and cell density, as well as enhanced virulence.^38,39^ Professor Xin Xu from the State Key Laboratory of Oral Diseases, West China School of Stomatology, Sichuan University gave a provoking talk regarding the role of *C. albicans* in the pathogenesis of root caries. They found that *C. albicans* was more abundant on root carious lesions than on healthy surfaces. Moreover, the role of *C. albicans* in cariogenicity was studied by using both in vitro and in vivo models. Data obtained from their group demonstrated the colonization of *C. albicans* could enhance the cariogenicity of oral biofilm, probably by upregulating the ratio of *Streptococcus mutans*/*Streptococcus sanguinis*. Additionally, *PHR2* was identified as a key factor that determines the successful colonization of *C. albicans* in a mixed microbial consortium under cariogenic condition, and thus may represent a promising target against root caries with Candida involvement.

Periodontitis is an oral infectious disease initiated by periodontal pathogens and influenced by host inflammatory and immune response, which results in progressive destruction of the periodontal tissues, including gingiva, periodontal ligament, and alveolar bone.^20,40–42^ Professor Denis Kinane from the University of Pennsylvania School of Dental Medicine gave an informative overview of the host responses to the microbial biofilms in periodontitis. The microbial biofilms, especially a triadic group of bacteria that comprises *Porphyromonas gingivalis*, *Treponema denticola* and *Tannerella forsythia*, are the initiators of periodontitis. The subsequent host response to microbial biofilm insult is through innate, inflammatory and adaptive immune responses. The extent and type of the host response are both individual- and bacteria-dependent. In addition, genetic variation and epigenetic variation were also associated with periodontitis susceptibility. Thus, an understanding of the interaction of structural and defensive host cells with the microbial plaque biofilm is pivotal to uncovering periodontal disease etiology and to developing tailored therapeutics.

In recent years, the concept that bacteria can be associated with cancer development has been well documented. *P. gingivalis*, a keystone pathogen in periodontal diseases, has been reported to be associated with oral squamous cell carcinoma (OSCC).^43–45^ Professor Yaping Pan from the School of Stomatology, Chinese Medical University delivered a wonderful lecture, which focused on their latest findings on *P. gingivalis* and its role in the development of OSCC. Studies from Pan’s group found that *P. gingivalis* was positively correlated with clinical stages, differentiation degree, and lymphatic metastasis of OSCC. Moreover, *P. gingivalis* was able to activate classic pathways involved in inflammation-associated tumorigenesis, possibly by inducing tumor-related genes such as *NNMT*, *FLI1*, *GAS6*, *lncRNACCAT1*, *PDCD1LG2*, and *CD274*. All these findings suggest that *P. gingivalis* could be a risk factor for the early development of OSCC and provide potential mechanism by which *P. gingivalis* can drive the progression of OSCC.

### Microbial biofilms and systemic diseases

The virulence of oral microbial biofilms is normally limited to the oral cavity. However, when these microbes or their components enter the connective tissues or circulation, they may increase the risk for some systemic diseases, such as cardiovascular diseases, diabetes, and immune-dysfunctional diseases.^21,25^ Inflammatory bowel disease (IBD) is an intestinal immune-dysfunctional disease worldwide whose prevalence is increasing in China as well as in Asia overall. Professor Feng Chen’s group investigated the structure and function of the oral microbiome in patients with IBD in order to identify new biomarkers for IBD. Their studies showed dysbiosis in the structure, composition, and function of oral microbiota associated with IBD, and indicated the potential for oral microbiota-based diagnosis and prognosis of IBD. More importantly, work from Dr. Chen’s group corroborates the recent findings reported by Atarashi et al. that strains of *Klebsiella spp*. from the salivary microbiota colonize in the gut and can potently induce chronic intestinal inflammation.^26^

## Promising approaches to the disruption of biofilms

The multifactorial nature of biofilm formation and drug resistance have imposed great challenges for the use of conventional antimicrobials.^46–49^ Just as our understanding of biofilm is evolving, technological advancements are providing unprecedented avenues to developing novel therapeutic methods that are promising for the disruption of biofilms.

Microbial cells in biofilms are embedded in an extracellular matrix consisting of polysaccharides, proteins, and extracellular DNA (eDNA) that functions as a “glue” to firmly attach them to surfaces and hold biofilm cells together, forming a cohesive and adherent ecosystem. Importantly, it creates a diffusion-modulating milieu that protects the resident microbes from antibiotics and helps them escape host immune systems.^1,5,9^ Thus, factors that disrupt the biofilm matrix may disassemble or disperse the biofilm and increase the sensitivity of biofilm bacteria to antibiotics, while facilitating its mechanical removal.^48^ Professor Lvyan Ma from the State Key Laboratory of Microbial Resources Institute of Microbiology, Chinese Academy of Sciences gave a talk titled “Biofilm disassembly and prevention by glycosyl hydrolase”, which highlighted how to combat biofilms by targeting the extracellular matrix. PslG, a self-produced glycosyl hydrolase by *Pseudomonas aeruginosa*, prevents biofilm formation and disassembles existing biofilms within minutes at nanomolar concentrations while supplied exogenously. PslG as an endoglycosidase mainly disrupts the Psl (a key biofilm matrix exopolysaccharide) to disperse bacteria from biofilms, consequently enhances markedly biofilm’s sensitivity to antibiotics and macrophage cells, resulting in improved biofilm clearance. Furthermore, PslG shows biofilm inhibition and disassembly activity against a wide range of Pseudomonas species, indicating its great potential in combating biofilm-related complications.

Advancements in the understanding of biofilms has brought forward a novel target for biofilm disruption. Since PNGase F was first discovered in *Elizabethkingia meningoseptica* in 1984, it has remained as the only *N*-glycosidase identified in bacteria.^50^ The talk “Bacterial *N*-glycosidase and its potential impacts on microbial−host interactions” by Professor Li Chen from the Key Laboratory of Medical Molecular Virology, Ministry of Education and Health, Fudan University introduced a newly identified *N*-glycosidase (PNGase F2) of *an E. meningoseptica* strain (FM007) isolated from a non-Hodgkin’s lymphomas (NHL) patient. This PNGase F2 is active on the substrates with core α-(1,3) fucose, indicating a potential role of *N*-glycosidase in microbial−host interactions. In addition, through a systemic functional genome analysis, a panel of glycosidase with novel activities were identified in the genome of FM007, and their potential impacts on cell surface modifications, cell−cell interactions, biofilm formation are currently under the investigation.

In the oral cavity, the diffusion-modifying properties of the extracellular matrix combined with metabolic activities of embedded microorganisms help create a highly acidic pH microenvironment, which not only initiates the demineralization of tooth hard tissues but also imposes environmental stress on the microbial community causing acid-sensitive species to perish and aciduric microbiota to flourish. Targeting the specific pathogenic microenvironments could be an attractive approach against cariogenic biofilms.^48^ Professor Hyun Koo from the University of Pennsylvania School of Dental Medicine focused his talk on this field and introduced new technologies using agents that are activated in response to low pH niches. Koo’s lab developed a new “smart” nanomaterial that triggers drug release in response to acidic pH, which enhances the antibiofilm activity of farnesol significantly improving its efficacy against caries severity in vivo (>10-fold) under a twice-daily topical treatment regimen. Another promising approach employs pH-responsive catalytic nanoparticles with antibiofilm and anticaries properties. These nanoparticles display peroxidase-like activity at acidic pH values found in cariogenic microenvironment, activating hydrogen peroxide in situ that simultaneously degrade the protective biofilm matrix barrier and kill the embedded bacteria for caries prevention without causing cytotoxicity in vivo. Antibiofilm technologies as exemplified above can be exploited for targeted therapy against biofilm-associated oral diseases and beyond.

## Microbiome-based oral disease prediction and single-cell approach

Big data is one of the most critical bottlenecks for microbiome-based precision medicine at present. By using a “BLAST-like” approach to comparing organismal/functional similarity of the query microbiome with the known microbiomes tagged with key metadata such as the type or stage of a polymicrobial disease or ecological disaster, a series of microbiome-based indices can be generated for the diagnosis or risk assessment of oral diseases.^51^

Over the past decade, group led by Professor Jian Xu from the Qingdao Institute of Bioenergy and Bioprocess Technology, Chinese Academy of Sciences has developed a series of microbiome-based metrics to quantitatively and objectively assess the health or “sub-optimal health” states of individual human hosts. These metrics are collectively called Microbial Indices of Health (MiHs), and include MiG (Microbial Index of Gingivitis),^52^ MiC (Microbial Indicator of Caries),^53^ MiSH (Microbial Index of Skin Health), etc. The first part of Professor Xu’s presentation introduced the ongoing projects on these fronts, in particular those projects that ask how these metrics can be applied to various age groups and population scales. Answers to these questions could form the basis for the employment of our microbiota in precise and preventive care, and for the development of new personal-care strategies or products.

However, despite rapid progress in microbiome-based modeling of human health, limitations of “meta-” approaches such as metagenome sequencing are evident. Three emerging trends in microbiome analysis platforms are: (i) expansion from dissecting microbiota structure by sequencing to tracking microbiota state, function, and intercellular interactions via function-based imaging; (ii) a shift from interrogation at the resolution of consortium or population of cells to that at the resolution of individual cells; and (iii) a change in the way of thinking from individual-project-based microbiome data analysis to a bird eye’s view of all microbiomes on Earth, i.e., the “microbiome data science”.^51^ Towards these goals, the Single-Cell Center have established a “Ramanome Technology Platform” (RTP).^54,55^ The second half of Professor Xu’s presentation explained the “Ramanome”/“Meta-ramanome” concept, which is a collection of single-cell Raman spectra (SCRS) acquired from individual cells within a cellular population or consortium. Professor Xu also introduced a suite of software called “Ramanome Software Trilogy” for automatic acquisition (RamLIS), high-throughput analysis (RamEX) and intelligent database-mining (RamDB) of such single-cell phenome data. In addition, Xu introduced a series of Raman-activated Cell Sorting (RACS) instruments that have been recently developed to physically sort individual cells of particular functions in a label-free manner for downstream single-cell sequencing or culturing. This new platform will make the functional validation of microbiome at single-cell resolution possible and will definitely advance our knowledge on the pathogenicity of specific microbes in a mixed consortium.

In addition to the speakers invited, this year’s symposium also included poster sessions from young investigators from China who presented the latest findings pertinent to microbial biofilms and the microbiome. The success of this year’s biofilm symposium showcased the breadth of PDM and SKLOD’s continued leadership roles in the generation of new knowledge and treatment modalities in preventing oral diseases, promoting oral health, and cultivating the next generation of academic leaders.

## Conclusions

The day ended with a group discussion focused on how to advance the biofilm field and accelerate the translation of basic research to clinical applications. Even though tremendous progress has been made in the last decade, particularly due to the introduction of the “microbiome” in this field, our understanding of the physiology and pathogenicity of microorganisms at genomic, transcriptomic and metagenomic levels that drive the pathogenesis of infectious diseases of human beings remain unanswered: What is the normal molecular baseline of human microbiota, particular for oral and gut microbiomes? What are the key factors driving the compositional and functional shift of the microbial community that can be exploited as potential target for the treatment of related diseases? How polymicrobial interactions occur within the confinement of the biofilm microenvironment? How the extracellular matrix and its components influences the localized microbial community to cause disease? How can we take full advantage of the rapid technological advances and translate it to the clinical treatment of oral and systemic infectious diseases? How can we apply and validate the oral microbiome-based disease prediction model at chair-side for susceptibility screening and prognosis prediction? Addressing these questions is an essential prerequisite to translating basic findings in microbiology to clinical applications. Because of the rapid progress of this field, symposium participants were in general agreement that an annual symposium bringing together top scientists and clinicians in the field to discuss the latest advancements and jointly tackling critical problems is necessary. A tentative schedule for the next joint symposium was decided for October 2018 to be held in Chengdu. We are expecting more new ideas to be generated from the upcoming event!
